# Supplementary material for: Association of Ct Values from Real-Time PCR with Culture in Microbiological Clearance Samples for Shiga Toxin-Producing Escherichia coli (STEC)
Source: Microorganisms. 2020 Nov 16;8(11):1801. doi: 10.3390/microorganisms8111801 (PMC7698137; doi:10.3390/microorganisms8111801)
Supplement: Supplementary file 1 [file microorganisms-08-01801-s001.pdf]

Supplementary Materials

Table S1. Patient Quadrant Growth and Corresponding Averaged Real-Time PCR Ct values.

Quadrant Growth	NEG[a]	1	2	3	4
Patient A Ct	33.48	N/A[b]	N/A	31.96	26.37
Patient B Ct	38.77	N/A	N/A	30.12	N/A
Patient C Ct	39.15	N/A	36.36	31.67	29.09
Patient D Ct	36.13	34.55	N/A	32.13	N/A
Patient E Ct	37.67	N/A	29.82	27.29	N/A
Patient F Ct	40	31	27.97	N/A	N/A
Patient G Ct	38.46	27.41	26.07	N/A	N/A
Patient H Ct	40	25	N/A	N/A	N/A
Patient I Ct	34.93	33.07	35.92	N/A	N/A
Patient J Ct	38.34	N/A	31.85	N/A	N/A
Patient K Ct	40	31.52	26.64	N/A	N/A
Patient L Ct	40	29.15	34.11	N/A	N/A
Patient M Ct	37.58	31.63	29.08	N/A	N/A
Patient N Ct	40	34.39	N/A	N/A	31.34

[a] Quadrant NEG indicates samples with no growth. [b] N/A indicate no sample received with growth in corresponding quadrant.
